# Supplementary material for: Splice-Junction-Based Mapping of Alternative Isoforms in the Human Proteome
Source: Cell Rep. Author manuscript; Available in PMC 2020 Jan 15. (PMC6961840; doi:10.1016/j.celrep.2019.11.026)
Supplement: 2 [file NIHMS1546469-supplement-2.zip › DF1/PXD010154/AdrenalGland/AdrenalGland_9_LYPLAL1_IIYPTAPPSYTVGPSFAR.pdf]

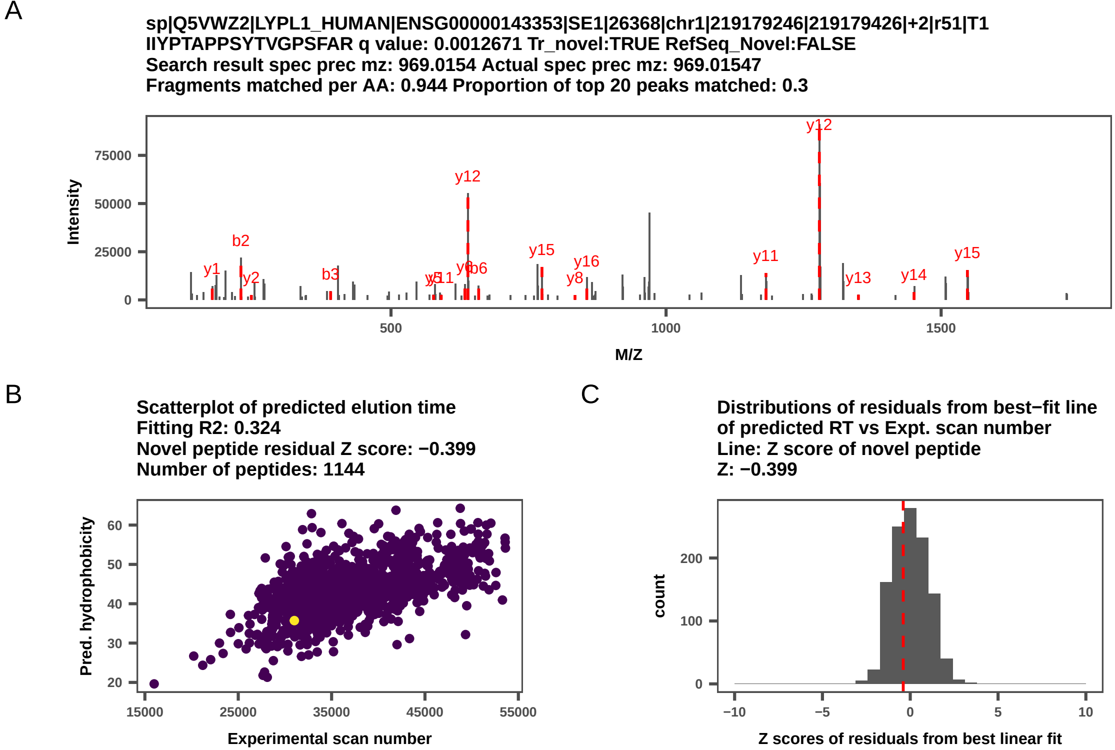

A
sp|Q5VWZ2|LYPL1_HUMAN|ENSG00000143353|SE1|26368|chr1|219179246|219179426|+2|r51|T1
IIYPTAPPSYTVGPSFAR q value: 0.0012671 Tr_novel:TRUE RefSeq_Novel:FALSE
Search result spec prec mz: 969.0154 Actual spec prec mz: 969.01547
Fragments matched per AA: 0.944 Proportion of top 20 peaks matched: 0.3
Intensity
75000
50000
25000
0
y1
b2
y2
b3
y12
y15
y16
y8
y11
y12
y13
y14
y15
500
1000
1500
M/Z
B
Scatterplot of predicted elution time
Fitting R2: 0.324
Novel peptide residual Z score: −0.399
Number of peptides: 1144
Pred. hydrophobicity
60
50
40
30
20
15000
25000
35000
45000
55000
Experimental scan number
C
Distributions of residuals from best−fit line
of predicted RT vs Expt. scan number
Line: Z score of novel peptide
Z: −0.399
count
200
100
0
−10
−5
0
5
10
Z scores of residuals from best linear fit
